# Structure Property Relationships of the Exotic Insulator–Insulator Transition in CeMnAsO_1–*x*_F_*x*_: A Potential Excitonic Insulator

**DOI:** 10.1021/acs.chemmater.6c00090

**Published:** 2026-04-02

**Authors:** S. Simpson, G. B. Lawrence, C. Ritter, E. J. Wildman, A. M. Arevalo-Lopez, K. Morita, Q. D. Gibson, A. C. Mclaughlin

**Affiliations:** † Department of Chemistry, 1019 University of Aberdeen, Meston Walk, Aberdeen AB24 3UE, U.K.; ‡ Department of Chemistry, 2707 University of Warwick, Gibbet Hill, Coventry CV4 7AL, U.K.; § Institut Laue Langevin, 71 Avenue des Martyrs, BP 156, Grenoble, Cedex 9 F-38042, France; ∥ Université de Lille, CNRS, Centrale Lille, ENSCL, Université d’Artois, UMR 8181-UCCS-Unitéde Catalyse et Chimie du Solide, Lille F-59000, France; ⊥ Department of Chemistry, 6572 University of Pennsylvania, Philadelphia, Pennsylvania 19104-6323, United States

## Abstract

A novel quantum insulator–insulator transition was recently reported upon F^–^ doping the Mott insulator CeMnAsO_1–*x*_F_*x*_. Below the transition, the resistivity increases by more than 2 orders of magnitude over a narrow temperature range, and a colossal Seebeck effect is observed alongside glassy dynamics. A combination of neutron diffraction, heat capacity, and transport measurements has enabled structure–property relationships of the exotic transition to be elucidated. Variable temperature high resolution neutron diffraction shows that there is no change in symmetry at the transition and that the transition can be controlled by increasing the electronic and magnetic coupling between the CeO/F and As–Mn–As blocks. Physical property measurements, combined with first-principles calculations, suggest that the transition is due to the formation of an unusual interlayer excitonic insulator state below the insulator–insulator transition, *T*_II_.

## Graphical abstract

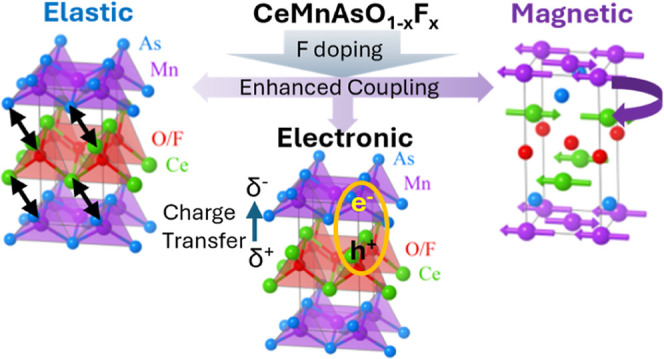

## Introduction

Layered pnictides are distinguished by the wealth of exotic physical phenomena they exhibit. Interest in these compounds was largely prompted by the discovery of high-temperature superconductivity (HTSC) in LaFeAsO_1–*x*_F_*x*_.^1,2^ Following this seminal work, HTSC was identified in several analogous layered pnictide materials, such as the 122-type BaFe_2_As_2_ family^3,4^ and 111-type LiFeAs compounds.^5,6^

Exchanging Fe for other 3d transition metals has resulted in the discovery of further interesting phenomena. For example, LnCoAsO displays itinerant ferromagnetism rather than HTSC,^7^ whereas *Ln*MnAsO phases (*Ln* = La, Nd) exhibit magnetoresistance (MR).^8^ MR is defined as a change in resistivity upon the application of a magnetic field H, such that MR = ((ρ_H_ – ρ_0_)/ρ_0_), where ρ_0_ and ρ_H_ are the resistivities in zero and applied field, respectively. Magnetoresistant materials find technological application in sensing and spintronics devices; discovering new magnetoresistant materials with enhanced properties represents an ongoing goal. The *Ln*MnAsO phases are Mott insulators, and charge transport is dominated by phonon-assisted hopping between localized states.^8^ Applying a magnetic field leads to destructive interference between direct and indirect hopping transitions, suppressing the localized nature of the charge carriers, increasing their contribution to the conductivity, and resulting in negative MR of up to −24% at 200 K.^8^

The MR properties of the *Ln*MnAsO series are extremely sensitive to chemical substitutions. For example, electron doping NdMnAsO by exchanging just 5% of the O^2–^ ions for F^–^ induces colossal magnetoresistance, such that MR_9 T_ (3 K) = −95%.^9^ PrMnAsO_0.95_F_0.05_ exhibits a structural transition from tetragonal to orthorhombic symmetry at 34 K driven by the Pr 4f electrons degrees of freedom, which results in a sizable MR_7T_ of −13.4% at 12 K.^10^ In contrast to electron doping, the hole-doped Nd_1–*x*_Sr_*x*_MnAsO series displays *positive* MR below 80 K,^11^ most likely due to a change in carrier mobility as reported for La­(Fe,Ru)­AsO.^12^

Recently, we reported a novel quantum insulating phase upon electron doping of the Mott insulator CeMnAsO. This phase is observed below a critical temperature *T*_II_ in the layered oxypnictide CeMnAsO_1–*x*_F_*x*_ (0.035 ≤ *x* ≤ 0.075), and *T*_II_ increases with F^–^ doping from 18 K (*x* = 0.035) to 82 K (*x* = 0.075). The same transition is also observed in Ce nonstoichiometric phases of the form Ce_*y*_MnAsO_0.95_F_0.05_ (*y* = 0.96, 0.97)^13^ with *T*_II_ = 104 and 50 K, respectively. The doping of F^–^ onto the O^2–^ site has a formally reducing effect on the compound, with an extra electron per substitution expected to fill the Mn–As layer-dominated conduction band. These electrons form a correlated insulating state (Mott insulator) upon cooling but retain some hopping conductivity. Surprisingly, neutron diffraction experiments and DFT calculations have shown that F^–^ substitution is also associated with an increase in the concentration of Ce^4+^, indicating formal oxidation of the Ce–O/F layer,^13^ so that the electronic structure of CeMnAsO_0.94_F_0.06_ is highly unusual.

Above *T*_II_, the samples are insulating and exhibit Anderson disorder. At the transition, their resistivities abruptly increase by more than 2 orders of magnitude over a narrow temperature interval and rapidly become immeasurably large. At the same time, a significant reduction in the electron mobility is observed alongside a colossal Seebeck effect. The transition is also accompanied by slow dynamics, which is thought to arise due to the decoupling of the electrons from lattice phonons.^13^ It was tentatively suggested that the insulator–insulator transition could be a result of many-body localization (MBL). However, it is not apparent yet if MBL is possible in greater than one-dimensional systems,^14–16^ and so, a more detailed investigation of the exotic quantum insulating state is needed.

In order to further explore this exotic insulator–insulator transition, we have performed a high-resolution neutron powder diffraction (NPD) study on CeMnAsO_1–*x*_F_*x*_ (*x* = 0–0.075) to elucidate important structure–property relationships across the series. We show that there is no change in crystal symmetry or magnetic structure at *T*_II_ and that the transition temperature can be tuned by both band gap engineering and reducing the Ce–As distance. Further analysis of refined bond lengths/angles, temperature-dependent thermal expansion, and heat capacity demonstrates that *T*_II_ is correlated with the structural, electronic, and magnetic coupling between the CeO/F layer and the As–Mn–As slab.

## Results and Discussion

### Neutron Diffraction

CeMnAsO_1–*x*_F_*x*_ crystallizes in the ZrCuSiAs structure type, which is described by the *P*4/*nmm* space group (Figure 1).^17^ To further explore structure–property relationships of the exotic insulator–insulator transition in CeMnAsO_1–*x*_F_*x*_, high-resolution NPD experiments have been performed on the solid solution CeMnAsO_1–*x*_F_*x*_ (*x* = 0–0.075) at 290 and 10 K. The same samples reported in ref 13 were used in the neutron diffraction experiments described here with *T*_II_ = 18 , 34, and 82 K for *x* = 0.035, 0.050, and 0.075, respectively. The samples are phase-pure apart from *x* = 0.075, which contains a minor CeOF impurity (∼1.5% by volume).

**Figure 1:**
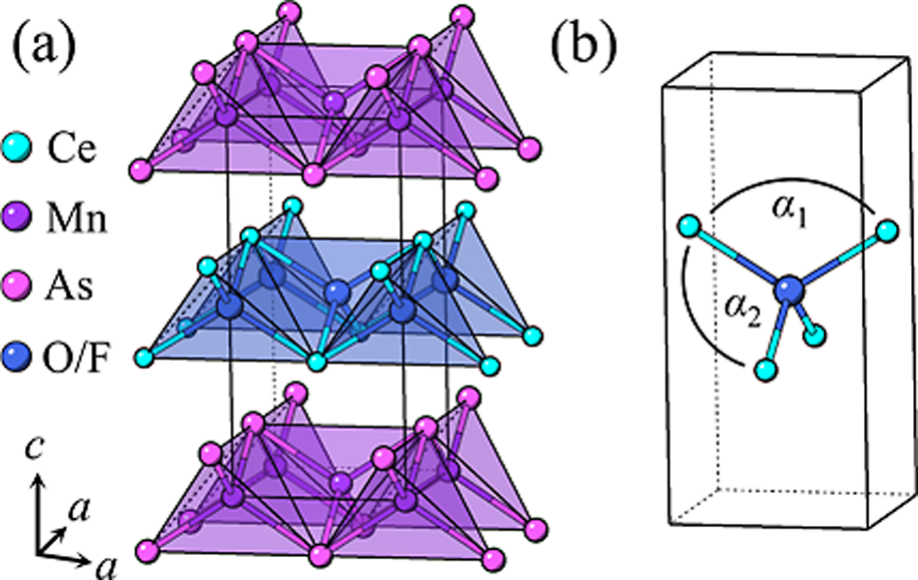
(a) Crystal structure of CeMnAsO_1–*x*_F_*x*_. (b) Isolated (O/F)­Ce_4_ tetrahedron with the two tetrahedral Ce–O/F–Ce angles α_1_ and α_2_ labeled.

The room temperature NPD patterns and corresponding Rietveld fits for CeMnAsO_1–*x*_F_*x*_ are shown in Figure 2. Cell parameters, agreement factors, and atomic parameters obtained from the refinements are provided in Supporting Information Tables 1 and 2. For the CeMnAsO_1–*x*_F_*x*_ phases, all atomic occupancies could be refined to within ±1% of their nominal values, so these were fixed for the remainder of the refinements. Cell parameters, agreement factors, and atomic parameters obtained from the refinements are provided in Section 1 of the Supporting Information.

**Figure 2:**
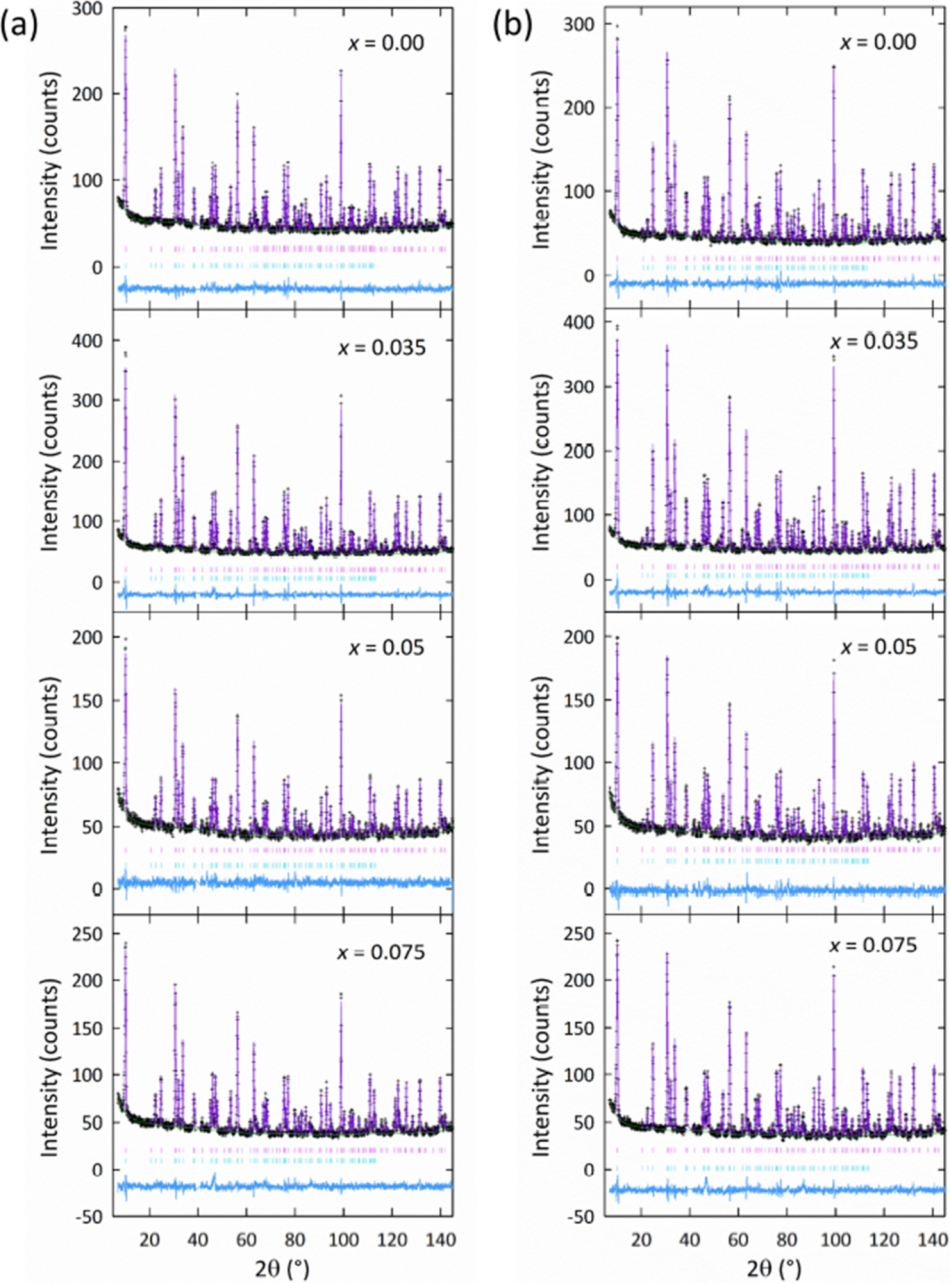
Rietveld fits with the *P*4/*nmm* structural model for the CeMnAsO_1–*x*_F_*x*_ series based on NPD data collected on D2B at (a) 290 K and (b) 10 K. The upper and lower tick marks in each plot correspond to the nuclear and magnetic phases, respectively.

Our refined structural models reveal several important correlations across the series. At 290 K, the Ce–O/F bond length expands from 2.3636(10) Å to 2.3670(12) Å as *x* increases from 0 to 0.075 (Supporting Information Figure 1), so that the interlayer Ce–As distance reduces upon increasing *x* in CeMnAsO_1–*x*_F_*x*_ (Figure 3) and the CeO/F layer moves closer to the semiconducting layer (As–Mn–As block). The same increase in the Ce–O/F bond length is observed in the superconducting CeFeAsO_1–*x*_F_*x*_ series^18^ and has been reported to facilitate electron transfer from the Ce–O/F layer to the As–Fe–As block, enhancing electronic coupling between the layers. There is no overall change in the Mn–As bond length or As–Mn–As angles upon increasing *x*. The Ce–(O/F)–Ce α_1_ bond angle decreases from 119.91(8)° to 119.58(10)°, as *x* increases from 0 to 0.075. At the same time, the Ce–(O/F)–Ce α_2_ bond angle increases from 104.52(4)° to 104.67(4)° (Supporting Information Figure 1). These values both tend slightly closer toward the ideal tetrahedral angle (109.47°) with increasing *x*.

**Figure 3:**
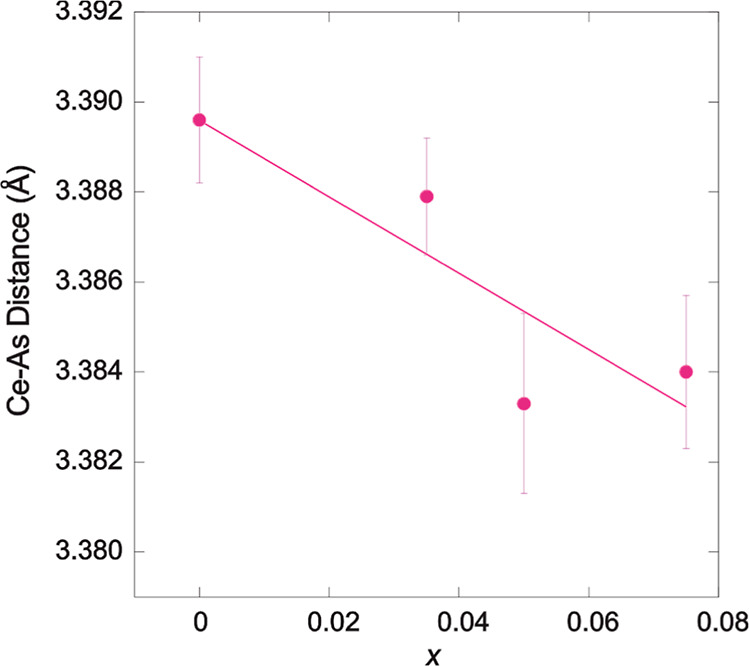
Variation of the Ce–As distance with F^–^ doping concentration, *x*, which shows that the effect of F^–^ doping is to move the Ce–O/F layer and the As–Mn–As block closer together.

Correlations between the tetrahedral shape and the physical properties of superconducting layered oxypnictides are well established. In particular, the superconducting transition temperatures, *T*_C_s, of CeFeAsO_1–*x*_F_*x*_ and SmFeAsO_1–*x*_F_*x*_ are enhanced, as the shape of the FeAs tetrahedra becomes more ideal and charge transfer to the FeAs layer is optimized.^18,19^ The variation of *T*_II_ with both α_1_ Ce–O/F–Ce and α_2_ Ce–O/F–Ce is shown in Figure 4, and there is a correlation between the two parameters, so that *T*_II_ is enhanced as the Ce tetrahedra become more ideal in the CeMnAsO_1–*x*_F_*x*_ series. This suggests that *T*_II_ is dependent on the interlayer charge transfer, so that there is increased electronic coupling between the Ce­(O/F) and MnAs layers upon doping with F^–^. We anticipate that further increases in *T*_II_ could be achievable by optimizing the Ce­(O/F) tetrahedral geometries, either as a consequence of chemical pressure within the Ce­(O/F) blocks or through the application of compressive strains.

**Figure 4:**
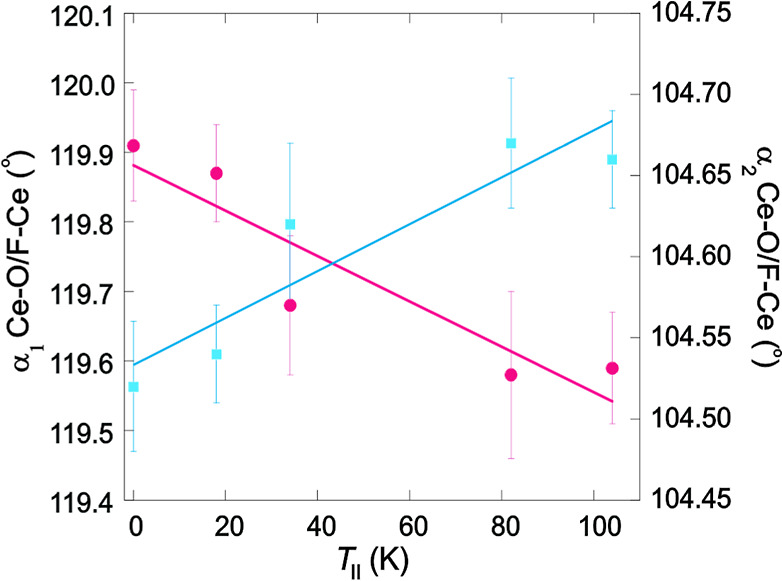
Variation of the 290 K α_1_ (pink circles) and α_2_ (blue squares) bond angles with *T*_II_.

There is no change in crystal symmetry upon cooling the samples, with the 10 K high-resolution neutron diffraction data also showing an excellent Rietveld fit to the structural model shown in Figure 2. We find no evidence of any subtle peak splitting or superstructure reflections within the high resolution of our diffraction experiments to suggest that there is any change in crystal symmetry upon cooling the samples from 290 K, with the ∼10 K neutron diffraction data showing an excellent Rietveld fit to the archetypical *P*4/*nmm* structural model (Figure 2). The Ce­(O/F) bond lengths still expand upon increasing *x* in the CeMnAsO_1–*x*_F_*x*_ series, so that the Ce–As distance reduces from 3.3779(13) Å for *x* = 0 to 3.3753(15) Å for *x* = 0.075 (Supporting Information Tables 3 and 4 and Figure 2). The MnAs layers do not display any clear trends upon varying *x*.

In CeMnAsO, the Mn^2+^ moments align antiferromagnetically below 367 K. The spins order antiferromagnetically in the *ab* plane but ferromagnetically along *c*, so that the magnetic structure can be indexed by the propagation vector *k* = (0, 0, 0)^13^ (C-type magnetic ordering). The Mn^2+^ moments align parallel to *c*, as shown in Figure 5a. Below 34 K, the Ce spins align antiferromagnetically with spins aligned parallel to the basal plane (Figure 5b). This results in a spin reorientation of the Mn^2+^ moments from aligning parallel to *c* to orienting parallel to the basal plane (Figure 5b). The refined magnetic moments are displayed in Supporting Information Tables 1 and 3. There is no change in the magnetic structure upon increasing *x* in CeMnAsO_1–*x*_F_*x*_. Below 10 K, the saturated Mn^2+^ magnetic moments differ by only ±0.01 μ_B_. In contrast, there is a significant reduction in the saturated Ce^3+/4+^ moment, which reduces from 1.16(4) μ_B_ for *x* = 0 to 0.99(5) μ_B_ for *x* = 0.075. This has been reported previously^13^ and is a result of partial oxidation of the Ce^3+^ spins upon substitution of O^2–^ with F^–^.

**Figure 5:**
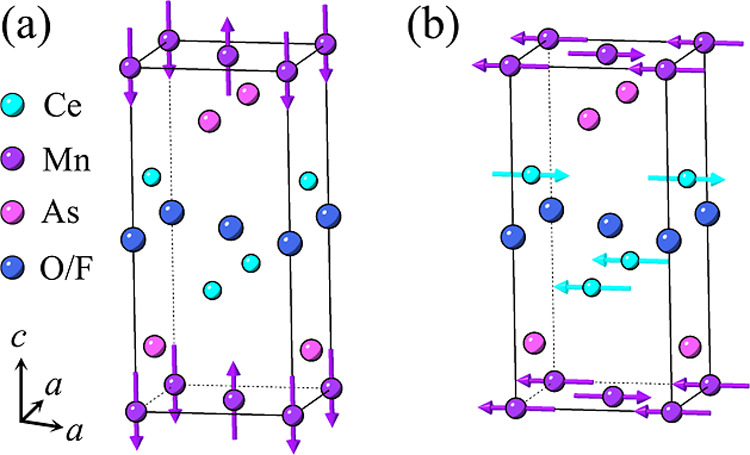
Magnetic structure of CeMnAsO_1–*x*_F_*x*_ at 290 K (a) and at *T* ≤ 34 K (b). The purple arrows relate to the antiferromagnetic order of the Mn^2+^ moments below 360 K, and the green spins relate to the antiferromagnetic order of the Ce^3+/4+^ moments below 34 K.

In order to investigate any possible magnetic anomalies at *T*_II_, variable temperature neutron diffraction patterns of CeMnAsO_1–*x*_F_*x*_ were recorded on the high intensity D20 diffractometer. Patterns were recorded between 1.5 and 380 K for *x* = 0.035 and 0.075 and between 300 and 380 K for *x* = 0.00 and 0.05 due to time constraints. A small reduction in the manganese magnetic transition temperature, *T*_Mn_, is observed from 367 to 359 K (Supporting Information Figure 3) upon F^–^ doping. Figure 6 shows that there is no significant change in the Ce magnetic transition (*T*_Ce_) or the spin reorientation temperature (*T*_SR_) upon increasing *x* from 0 to 0.075 in CeMnAsO_1–*x*_F_*x*_. The spin reorientation transition occurs over a 10 K region, with complete rearrangement of Mn^2+^ by 27 K in both samples. This is in agreement with the magnetic susceptibility data previously reported for CeMnAsO_1–*x*_F_*x*_.^13^ There is also no significant change in the magnetic structure or the magnitude of the Mn^2+^ or Ce^3+/4+^ moments at *T*_II_, so that the transition to the novel quantum insulating phase in CeMnAsO_1–*x*_F_*x*_ (*x* > 0.03) does not have a magnetic origin.

**Figure 6:**
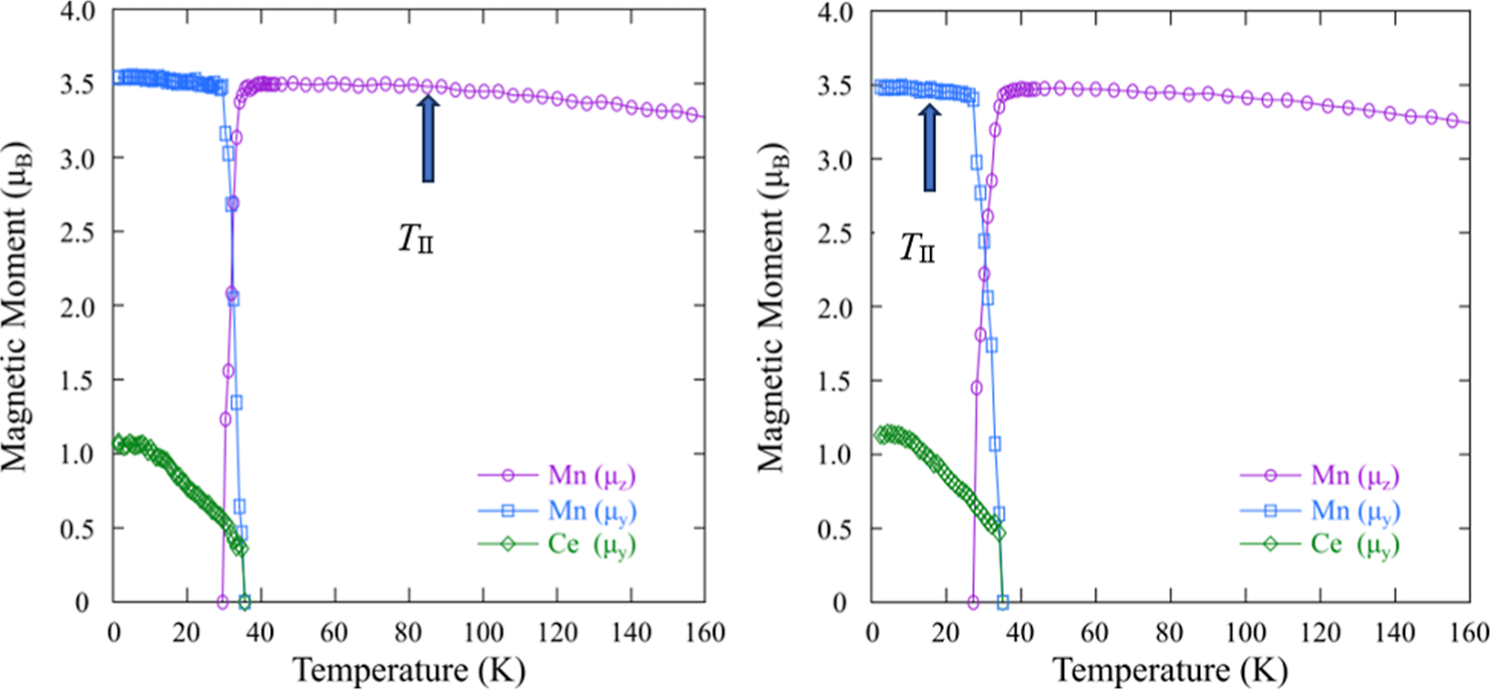
Temperature variation of the refined Ce^3+/4+^ and Mn^2+^ moments for CeMnAsO_0.925_F_0.075_ (left) and CeMnAsO_0.965_F_0.035_ (right) using neutron diffraction patterns from the high intensity D20 diffractometer. The arrow highlights *T*_II_ = 82 and 18 K, respectively. There is no significant change in the Ce magnetic transition (*T*_Ce_) or the spin reorientation temperature (*T*_SR_) upon increasing *x* from 0 to 0.075 in CeMnAsO_1–*x*_F_*x*_ within the error of the neutron diffraction data.

The temperature variation of the lattice parameters was determined from Rietveld fits to the *P*4/*nmm* structural model from the D20 neutron diffraction data for the CeFeAsO_1–*x*_F_*x*_ series (Supporting Information Figure 4). The linear coefficients of thermal expansion were calculated by fitting the temperature dependence of the lattice parameters to a third-order polynomial, separately from 50 K to 300 K and 10 K to 30 K (above and below *T*_SR_), and are shown for CeMnAsO_0.965_F_0.035_ in Figure 7. The coefficients of thermal expansion all begin to plateau above 200 K, consistent with the Debye temperature of 230(10) K (calculated from the heat capacity as described below and in Supporting Information Section 3).

**Figure 7:**
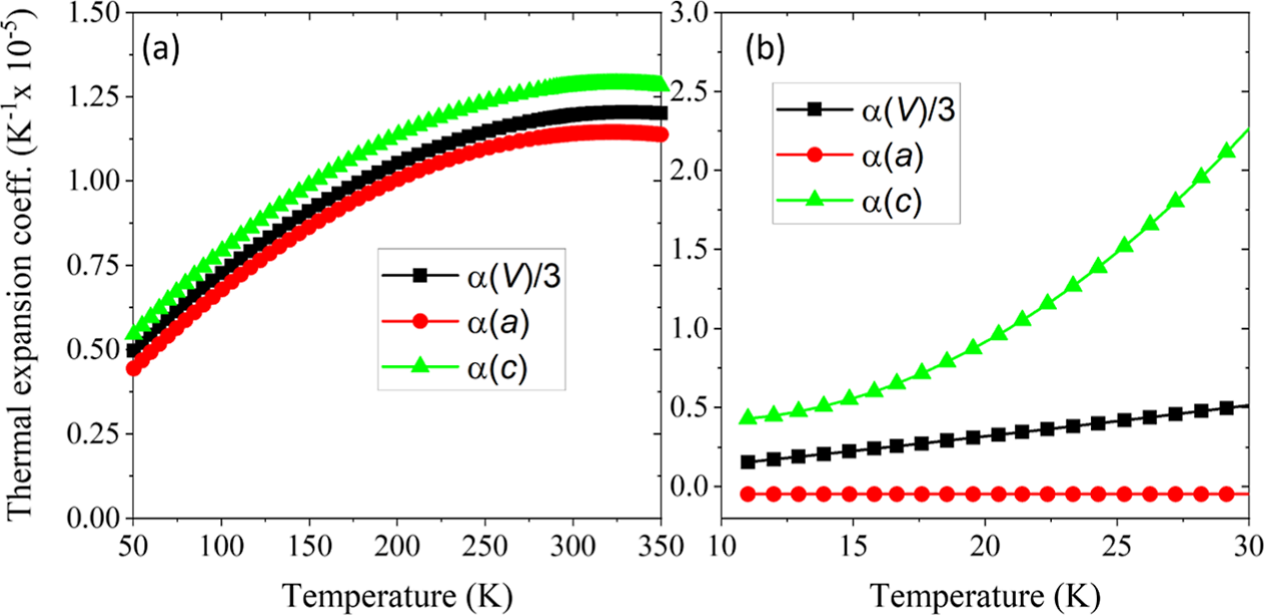
Linear coefficients of thermal expansion for *a* (red), *c* (green), and cell volume (black) for CeMnAsO_0.965_F_0.035_. CeMnAsO_1–*x*_F_*x*_ phases exhibit a spin reorientation of the Mn^2+^ moments below *T*_SR_ = 34 K, and so the data are shown for the temperature ranges 50 K–350 K (above *T*_SR_) (a) and 10 K–30 K (below *T*_SR_) (Supporting Information Section 2) (b). The cell volume data are divided by a factor of 3 for a more direct comparison.

α­(*a*) is approximately zero below 30 K, whereas *c* appears to plateau at a constant (finite) value. Below *T*_SR_, the elastic properties are anisotropic due to the magnetostriction observed in *c* (Supporting Information Figure 4), which can be attributed to the interlayer magnetic coupling between the Mn^2+^ spins and Ce^3+^ spins.^20^ Unusually, at 300 K (well above *T*_SR_), the anisotropy ratio α­(*c*)/α­(*a*) increases significantly from 1.08(3) to 1.20(3) as *x* is changed from 0.00 to 0.075.

Figure 8a shows that the anisotropy in the thermal expansion increases as the Ce–As distance, and therefore the interlayer distance, reduces. Reducing the Ce–As distance facilitates charge transfer, so that fluorine doping enhances electronic coupling between the Ce­(O/F) and MnAs layers, as already evidenced by the correlation between *T*_II_ and with both α1 Ce–O/F–Ce and α2 Ce–O/F–Ce bond angles (Figure 4). The resulting change in anisotropy ratio with chemical doping reflects this stronger interlayer coupling, making it a quantitative proxy for the interlayer coupling strength between the CeO/F layer and the As–Mn–As block.

**Figure 8:**
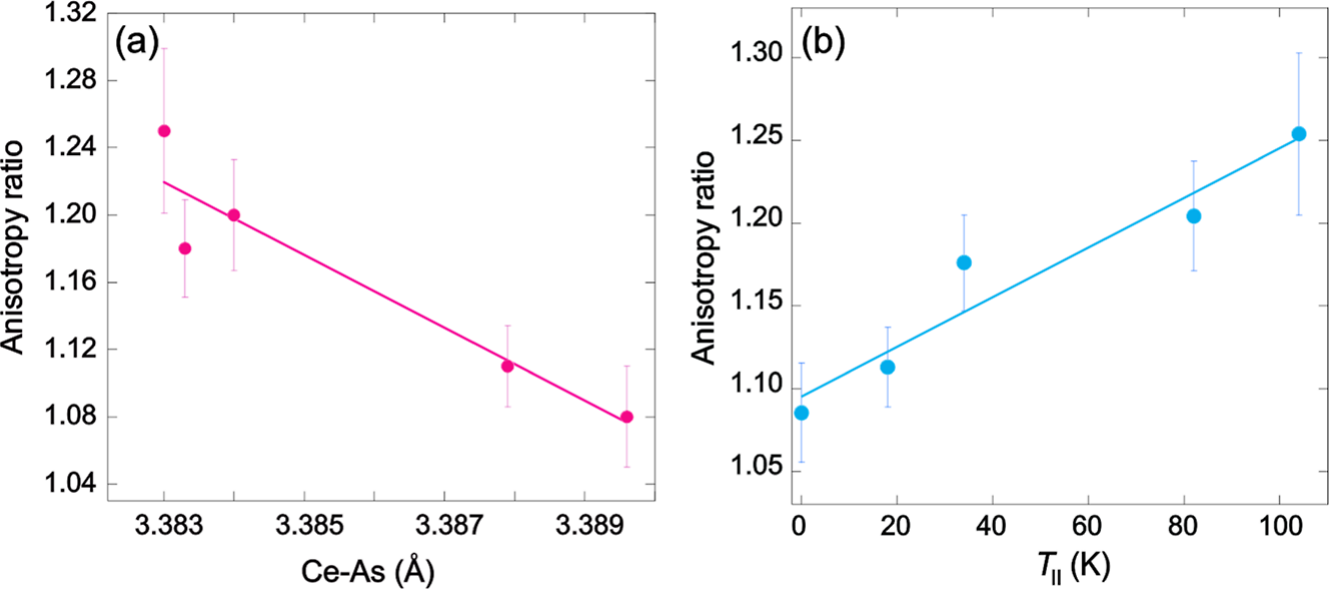
Coupling of the electronic and structural degrees of freedom in CeMnAsO_1–*x*_F_*x*_ and Ce_*y*_MnAsO_0.95_F_0.05_. (a) Variation of the anisotropy ratio with the Ce–As distance, showing that the anisotropy ratio is enhanced as the Ce–As distance decreases. (b) Direct relationship between the anisotropy ratio and *T*_II_. Both plots show the data for CeMnAsO_1–*x*_F_*x*_ (*x* = 0.03–0.075) and Ce_0.96_MnAsO_0.95_F_0.05_.

We have previously shown that *T*_II_ can also be tuned by varying the Ce nonstoichiometry in Ce_*y*_MnAsO_0.95_F_0.05_, where *T*_II_ = 34 and 104 K for *y* = 0.00 and 0.96, respectively.^13^ Figure 8b shows that there is a clear correlation between the anisotropy ratio and *T*_II_ across all CeMnAsO_1–*x*_F_*x*_ and Ce_0.96_MnAsO_0.95_F_0.05_ phases reported here, so that *T*_II_ monotonically increases as the interlayer electronic coupling is enhanced. Hence, the significant enhancement of *T*_II_ with either F^–^ doping or Ce non-stoichiometry is related to the increased interlayer electronic coupling.

### Heat Capacity

The coupling between the CeO/F layer and the As–Mn–As block is further evident in heat capacity measurements, where a low temperature Schottky contribution to the heat capacity is observed below *T*_SR_ (Figure 9). This has previously been reported for CeMnAsO.^20^ This Schottky contribution is crucial for modeling the temperature dependence of *C*_P_/*T* at temperatures below *T*_SR_; a more than 50% contribution to the shape is observed when *T* < 20 K, and the shape of the Schottky contribution is highly dependent on the splitting energy. The splitting energies were modeled as *E*_S_/*k*_B_ = 41 K, 45(1) K, and 48(2) K for CeMnAsO,^20^ CeMnAsO_0.965_F_0.035_ (*T*_II_ = 18 K), and Ce_0.96_MnAsO_0.95_F_0.05_ (*T*_II_ = 104 K), respectively (Supporting Information Section 3, Figure 9 and Supporting Information Figure 5). Errors were estimated by the amount of change in the parameter needed to affect the quality of the fit. The number of Schottky states per formula unit in the model also decreases from 0.21 in CeMnAsO_0.965_F_0.035_ (*T*_II_ = 18 K) to 0.19 in Ce_0.96_MnAsO_0.95_F_0.05_ (*T*_II_ = 104 K), consistent with the increased concentration of Ce^4+^ from neutron diffraction data. Only very minor changes to modeling the phonon contribution to the heat capacity were used; the details of the full heat capacity models for these materials are shown in Supporting Information Table 5 and Figure 5. The Schottky contribution arises from the splitting of the ground state doublet of 4f^1^ Ce^3+^ due to the change in exchange coupling when the Mn^2+^ moments reorientate, as previously reported at *T*_SR_ in CeCrAsO^21^ and at *T*_N_ for CeFeAsO.^22^ The increase in the splitting energy upon increasing *x* in CeMnAsO_1–*x*_F_*x*_ suggests enhanced magnetic exchange between Ce^3+^ and Mn^2+^ with increased F^–^ doping as the interlayer Ce–As distance is reduced and the electronic and structural coupling between layers is enhanced.

**Figure 9:**
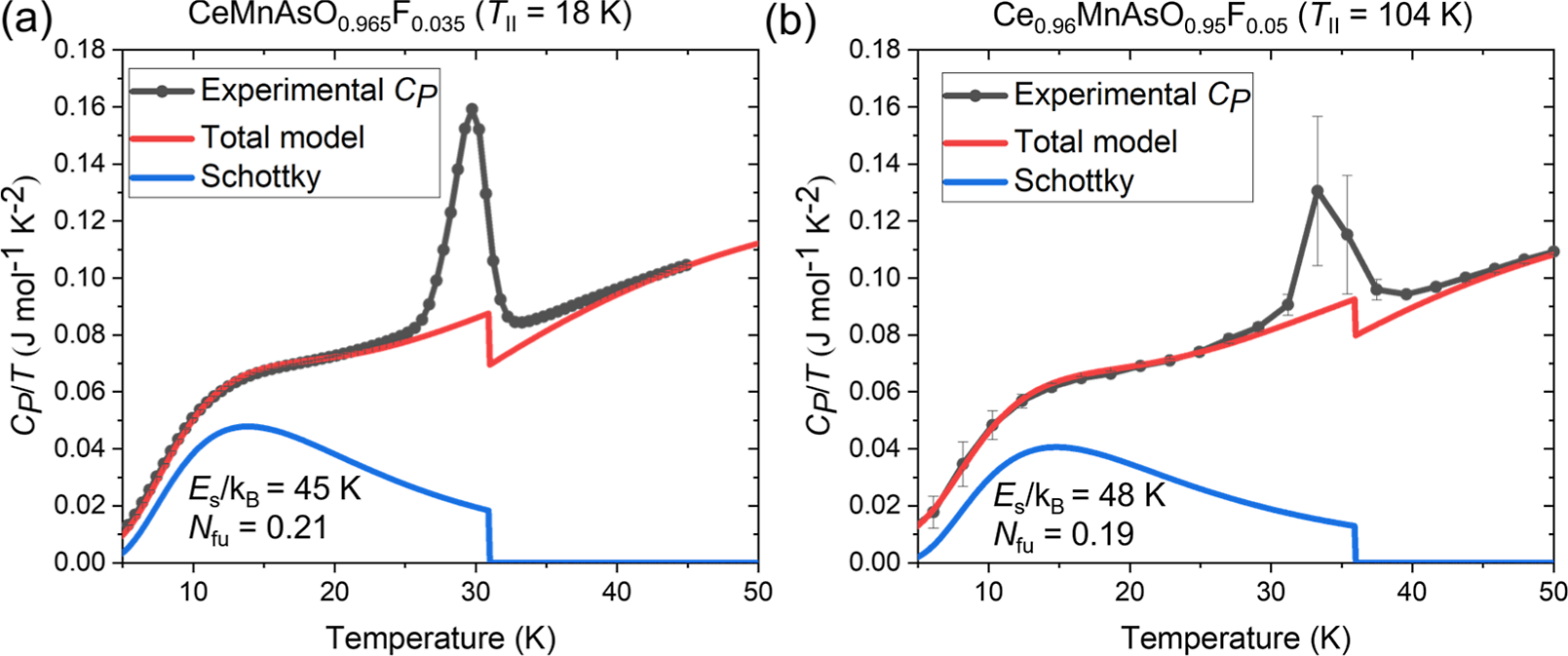
Low temperature plots of *C*_P_/*T* against temperature for (a) CeMnAsO_0.965_F_0.035_ and (b) Ce_0.96_MnAsO_0.95_F_0.05_, highlighting the Schottky contribution, the total heat capacity model, and the difference in splitting energy (*E*_S_/*k*_B_) and defect concentration (*N*_fu_). Error bars as standard deviations of the average of three data points for every temperature are shown.

### Electronic Properties

Neutron diffraction experiments and DFT calculations have previously shown that the electronic structure of CeMnAsO_0.94_F_0.06_ is highly unusual.^13^ Upon electron doping CeMnAsO, by substitution of F^–^ for O^2–^, the Ce 4f band is destabilized in the vicinity of F^–^, so that upon F^–^ doping, some of the Ce^3+^ oxidizes to Ce^4+^, hence creating holes in the 4f band. At the same time, there is reduction in the Mn 3d band by the electrons added by substitution of F^–^ for O^2–^ and the simultaneous oxidation of Ce^3+^. To explore this further, Hall coefficient measurements were performed on Ce_0.97_MnAsO_0.95_F_0.05_ with *T*_II_ = 50 K, where the presence of both electron and hole carriers is observed (Figure 10).

**Figure 10:**
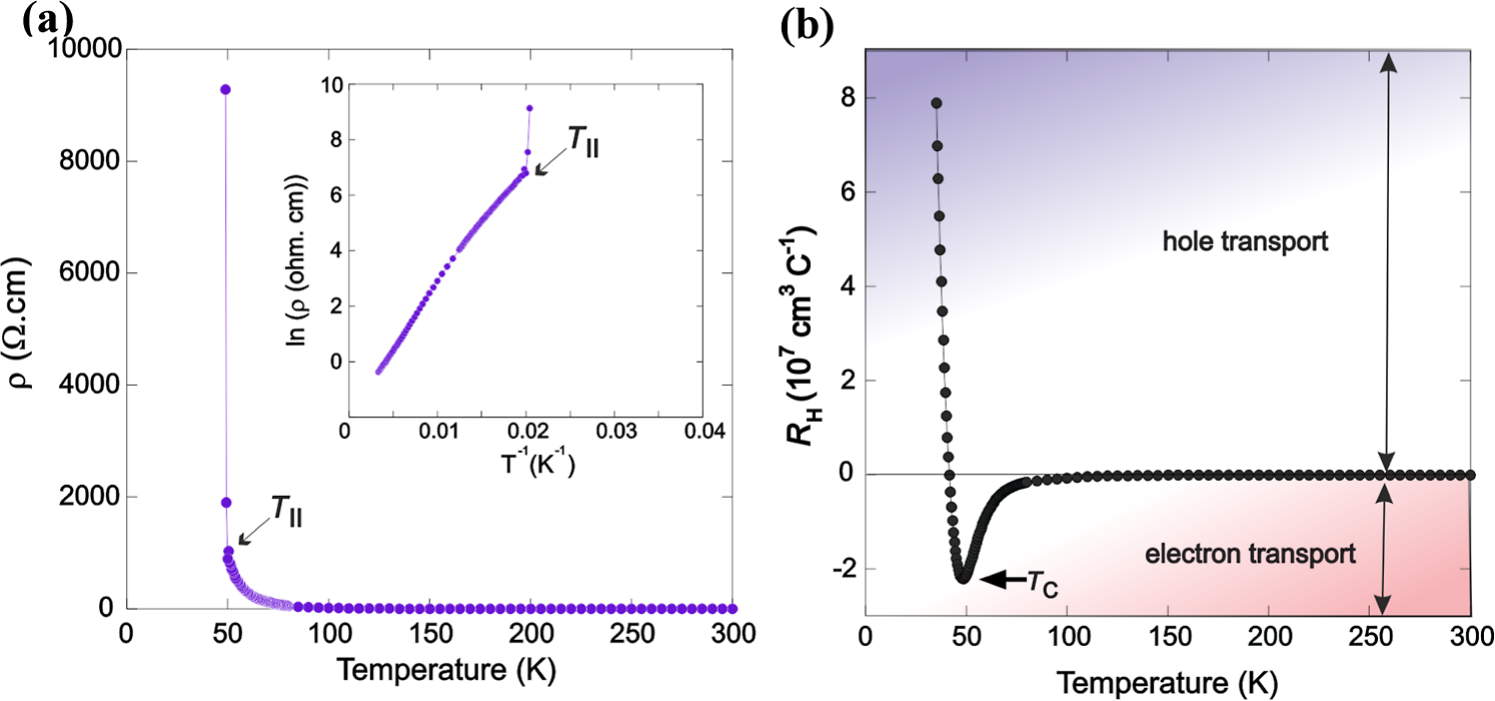
Electronic properties of Ce_0.97_MnAsO_0.95_F_0.05_. (a) Temperature variation of the resistivity of Ce_0.97_MnAsO_0.95_F_0.05_, evidencing an insulator–insulator transition at *T*_II_ = 50 K. The inset shows the temperature dependence of ln­(ρ), where a clear divergence in ln­(ρ) is observed at *T*_II_. (b) Temperature variation of the Hall coefficient for Ce_0.97_MnAsO_0.95_F_0.05_, which has a *T*_II_ of 50 K. Results show a sign reversal from *R*_H_ being negative to positive below *T*_II_. This suggests that the remaining dominant carriers change from electrons to holes as the excitons condense.

The Hall coefficient (*R*_H_) in the presence of both electrons and holes can be described by

$$R_{H}=\frac{{\mu_{h}}^{2}p-{\mu_{n}}^{2}n}{e{\left(\mu_{h}p+\mu_{n}n\right)}^{2}}$$

where μ_h_ and μ_n_ are the hole and electron mobilities, and p and n are the hole and electron concentrations. In the event of roughly equal hole and electron concentrations, the sign of the Hall coefficient will be dominated by the carrier type that has the larger mobility. In this case, the negative Hall coefficient above *T*_II_ and positive coefficient below *T*_II_ are consistent with a scenario of heavy, nearly immobile holes, and lighter, more mobile electrons above *T*_II_, with both carriers freezing out at *T*_II._

The crossover from a negative Seebeck coefficient to a positive Seebeck coefficient below *T*_II_ = 18 K for CeMnAsO_0.965_F_0.035_ ^13^ is also consistent with this picture. The Seebeck effect is largest at very low carrier concentrations of noncompensated carriers. Thus, if the electrons and holes freeze at *T*_II_, the remaining carriers simply represent a small number of whatever electrons and holes are left over due to small imbalances of the chemical potential. The Seebeck coefficient will then be very large and either positive or negative, depending on details of the defect chemistry, consistent with what has been observed.^13^ Hence, it would appear that electrons dominate both the Hall and Seebeck response above *T*_II_. A crossover from a negative to a positive Hall coefficient (and a negative to positive Seebeck coefficient) is observed below *T*_II_, which suggests that an extremely low density of free charge carriers (holes) remains below the insulator–insulator transition.

One scenario to explain the freezing out of both holes and electrons below *T*_II_ in CeMnAsO_1–*x*_F_*x*_ is the formation of an excitonic insulator (EI) state. EIs are a remarkable class of insulators that can exhibit a condensate of electron–hole pairs (excitons) below a critical temperature and are predicted to give rise to exotic quantum phenomena such as superfluid energy transport alongside exotic quantum phases of excitons.^23,24^ A new approach to realizing the EI phase has been to construct charge transfer interlayer excitons by separating the electrons and holes in distinct layers. A correlated interlayer EI has recently been reported in heterostructures of monolayer WSe_2_ and moiré WS_2_/WSe_2_.^25,26^ Here, interlayer excitons form when electrons are added to the Mott insulator in the WS_2_/WSe_2_ bilayer, and holes are added to the WSe_2_ monolayer under an out-of-plane electric field. Excitonic condensation was observed below 60 K. By analogy to this, we hypothesize that the pairing of heavy holes from the CeO/F layer and electrons from the Mott-insulating MnAs layer could enable similar interlayer excitons to emerge below *T*_II_ in CeMnAsO_1–*x*_F_*x*_ (Figure 11).

**Figure 11:**
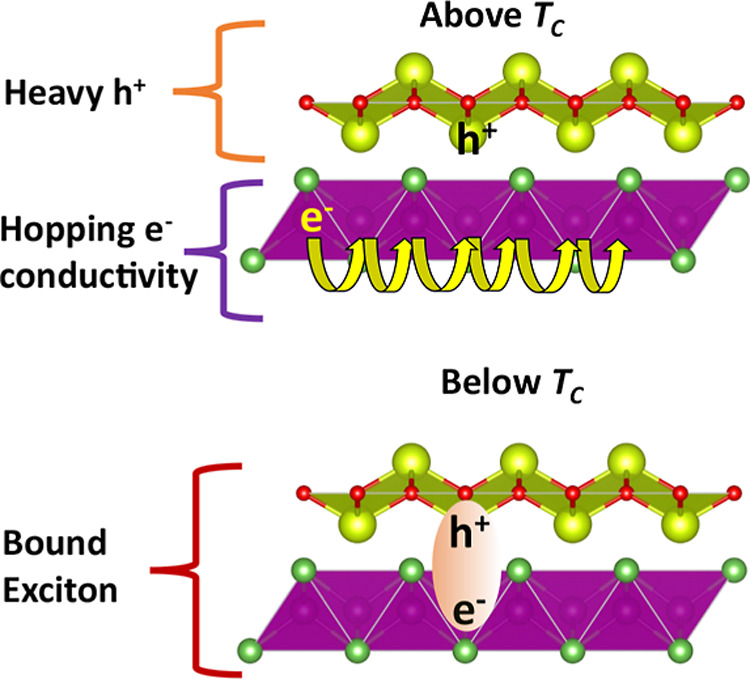
Exciton formation in CeMnAsO_1–*x*_F_*x*_. A schematic of the hypothesized formation of the correlated interlayer excitonic insulator phase in CeMnAsO_1–*x*_F_*x*_ below *T*_II_, where holes from the CeO/F layer bind with electrons from the Mott insulating As–Mn–As block.

### First Principles Calculations

To investigate if excitons are supported in CeMnAsO, first-principles calculations were performed. Using density functional theory (DFT) Kohn–Sham single-particle wave function as a starting point, we utilized the G_0_W_0_ method to calculate the band structure. The obtained band gap was 1.0 eV, which was larger than the experimental electronic band gap, so we applied scissors correction to match the experimental band gap of 0.31 eV. The resulting band structure is presented in Figure 12a. Although the *S*-point had a slightly narrower band gap, the band structure indicates that a Γ–Γ exciton can be hosted. To characterize excitons, based on the G_0_W_0_ method, we performed the Bethe–Salpeter equation (BSE) calculation. The optical response function and joint density of states are presented in Figure 12b. Two exciton peaks are clearly discernible around 0.27 and 0.31 eV, which correspond to exciton binding energies *E*_b_ = 0.05 and 0.01 eV, respectively, supporting the presence of interlayer excitons in CeMnAsO.

**Figure 12:**
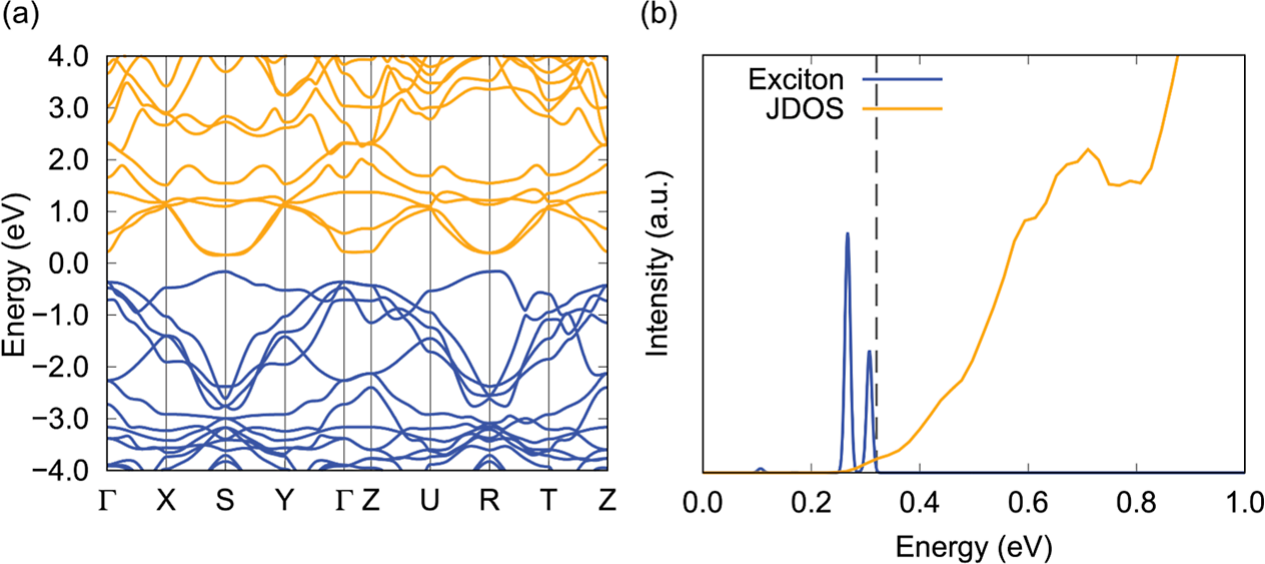
Calculated electronic properties of CeMnAsO. (a) Electronic band structure of CeMnAsO calculated by the G_0_W_0_ method. The conduction and the valence band are colored in yellow and blue, respectively. The energy was shifted, so that 0.0 eV corresponds to the middle of the band gap. (b) Exciton states calculated by the BSE and joint density of states calculated by the G_0_W_0_ method.

Combined, our structure–property relationships, physical property measurements, and first-principles calculations are consistent with the emergence of an interlayer EI below *T*_II_ in CeMnAsO_1–*x*_F_*x*_. It has been previously shown that there is an optimum interlayer distance for observing the interlayer EI phase.^27^ Reducing the Ce–As distance and increasing interlayer coupling will result in a greater spatial overlap between the electrons and holes and hence enhance the interlayer Coulomb interaction, leading to a greater exciton binding energy (*E*_b_) and higher transition temperatures, as observed here.^27,28^

On the semiconducting side of the EI phase diagram, a transition from a semiconductor to an EI is observed when the bandgap, *E*_g_, is reduced to a critical level (so that binding energy of the exciton, *E*_b_, is greater than *E*_g_). The transition temperature from the semiconductor to the EI phase is also observed to increase, as *E*_g_ is further reduced, reaching a maximum at *E*_g_ = 0 eV. Our combined experimental and theoretical insights allow us to construct an electronic phase diagram of the insulator–insulator transition in CeMnAsO_1–*x*_F_*x*_ and compare it against the canonical EI phase diagram (Supporting Information Figure 6). CeMnAsO is a Mott insulator; substituting F^–^ for O^2–^ in CeMnAsO_1–*x*_F_*x*_ reduces the electronic bandgap, *E*_g_, by almost an order of magnitude from 0.31(5) eV to 0.04(2) eV.^13^ Upon cooling, CeMnAsO_1–*x*_F_*x*_ phases with *x* ≥ 0.035 undergo a transition into a quantum insulating phase below a critical temperature *T*_II_. The emergence of an insulating phase, only when *E*_g_ is tuned below a critical threshold by carrier doping, alongside a *T*_II_ that increases from 18 K (*x* = 0.035) to 82 K (*x* = 0.075), as the system approaches the semi metallic regime (Supporting Information Figure 6b), is consistent with the theoretical expectations for EI formation (Supporting Information Figure 6a), giving further evidence that the quantum insulating phase observed in CeMnAsO_1–*x*_F_*x*_ could be the result of a transition to an EI below *T*_II_.

## Conclusions

We have elucidated crucial deeper insight regarding the origins of the exotic insulator–insulator phase in CeMnAsO_1–*x*_F_*x*_. Although there is no change in the crystal or magnetic structure at *T*_II_, our high-resolution neutron diffraction and heat capacity studies show that the electronic and magnetic coupling between the CeO/F layer and the As–Mn–As block layers increases with *x*, so that *T*_II_ can be tuned by both band gap engineering and by modifying the interlayer distance. Our combined experimental and theoretical evidence suggests that CeMnAsO_1–*x*_F_*x*_ may be a tunable correlated interlayer EI below *T*_II_.

Above *T*_II_ in CeMnAsO_1–*x*_F_*x*_, the charge carriers are localized as a result of Anderson localization, and variable range hopping is observed.^13^ Hence, the electrons are localized, and electron transport is only possible by phonon-assisted tunneling between localized states. Below *T*_II_, the presence of the exciton condensate and disorder would most likely block all thermalization channels, which would explain the gradual decoupling of electrons and phonons in CeMnAsO_1–*x*_F_*x*_ and the significant reduction in Hall mobility.^13^ The residual charge carriers are then deeply localized (potentially many-body localized), so that the resistivity rapidly increases and becomes immeasurable below *T*_II_.

The discovery of a novel correlated interlayer EI phase in CeMnAsO_1–*x*_F_*x*_ would be important, as EI phases are predicted to exhibit exotic properties and have technological applications such as in excitonic transistors and switches. CeMnAsO_1–*x*_F_*x*_ is hence a promising chemical platform for further investigating the exotic quantum phases of excitons. Further experiments to verify the presence of excitons in CeMnAsO_1–*x*_F_*x*_ are now warranted.

## Methods

### Synthesis

Polycrystalline samples of CeMnAsO_1–*x*_F_*x*_ (*x* = 0.00, 0.035, 0.05, 0.075) were prepared in a two-step solid-state reaction. A CeAs precursor was first synthesized by reacting stoichiometric quantities of Ce pieces (Aldrich 99.9%) and As chips (Alfa Aesar 99.999%) in an evacuated quartz tube at 980 °C for 33 h. This precursor was then reacted with stoichiometric masses of Mn, MnO_2_, and MnF_2_ powders before being ground in an agate mortar and pestle under an inert atmosphere. The powder was then pressed into pellets using a 10 mm die set, placed in a Ta crucible, and sealed in an evacuated quartz tube before being sintered at 1150 °C for 48 h.

### Diffraction

Laboratory X-ray powder diffraction (XRD) patterns were collected on a PANalytical Empyrean powder diffractometer equipped with a Cu Kα tube at ambient temperature. Data were recorded in the range 10° < 2θ < 110° with a step size of 0.013°.

NPD measurements were recorded on the high-resolution D2B diffractometer at the Institut Laue-Langevin (ILL, Grenoble) at a constant wavelength of 1.594 Å. Data were recorded at 1.5–10 and 300 K for ∼0.6 g samples of CeMnAsO_1–*x*_F_*x*_ in an 8 mm vanadium can, with a collection time of 4 h for each sample at each temperature. Due to issues with the cryostat, the low-temperature recordings were not all at 1.5 K; each different doping level was recorded at a slightly different temperature (all temperatures were ≤10 K). High-intensity neutron diffraction data were recorded on the D20 beamline between 1.5 and 380 K for *x* = 0.035 and 0.075 and between 300 and 380 K for *x* = 0.00 and 0.05. The ramp rates for data recording were 19 s per 0.1°. Rietveld refinements^29^ were performed using the GSAS/EXPGUI package^30^ to determine both the nuclear and magnetic structures of the series at the two temperatures investigated. Data were excluded in the range 39–41° 2θ in all the refinements performed due to peaks from the cryostat, a consequence of the small sample mass. The backgrounds were fitted using a shifted Chebyschev function, and the peak shapes were modeled with a pseudo-Voigt function.

### Physical Property Measurements

The temperature dependence of the Hall resistivity, electronic resistivity, and Seebeck effect of the selected CeMnAsO_1–*x*_F_*x*_ phases was recorded using a Quantum Design physical property measurement system (PPMS) between 4 and 300 K.

### First-Principles Calculations

The electronic structure was calculated using plane-wave DFT within the projector-augmented wave scheme as implemented in VASP. Following our previous work,^13^ a cutoff energy of 600 eV and reciprocal space sampling of 6 × 6 × 3 were used.^31–33^ Initial DFT calculation was performed with PBE + *U* functional within the collinear spin representation, where Hubbard *U* was set to 5.0 and 1.0 eV for Ce-f and Mn-d orbitals, respectively.^34,35^ G_0_W_0_ was performed on top of the single-particle wave functions. The plane-wave cutoff energy was 400 eV used both for wave functions and the response function. Twelve occupied and 12 unoccupied states were selected and used to calculate the BSE calculation with Tamm–Dancoff approximation.^36^

## Supplementary Material



## Notes

Crystallographic data, detailed information on the heat capacity modeling, and heat capacity data (PDF)
