# Supplementary material for: Structure Property Relationships of the Exotic Insulator–Insulator Transition in CeMnAsO1–x F x : A Potential Excitonic Insulator
Source: Chem Mater. 2026 Apr 2;38(7):3630–8. doi: 10.1021/acs.chemmater.6c00090 (PMC13085232; doi:10.1021/acs.chemmater.6c00090)
Supplement: Supplementary file 1 [file cm6c00090_si_001.pdf]

Supplementary data for:

Structure Property Relationships of the Exotic Insulator-Insulator Transition in $CeMnAsO_{1-x}F_x$: A Potential Excitonic Insulator

S. Simpson [1, 2], G. B. Lawrence [1], C. Ritter [3], E. J. Wildman [1], A. M. Arevalo-Lopez [4], K. Morita [5], Q. D. Gibson [1] and A. C. Mclaughlin [1*]

[1] Department of Chemistry, University of Aberdeen, Meston Walk, Aberdeen, AB24 3UE, U.K.

[2] Department of Chemistry, University of Warwick, Gibbet Hill, CV4 7AL, Coventry, U.K

[3] Institut Laue Langevin, 71 Avenue des Martyrs, BP 156, F-38042 Grenoble Cedex 9, France.

[4] Université de Lille, CNRS, Centrale Lille, ENSCL, Université d'Artois, UMR 8181-UCCS-Unitéde Catalyse et Chimie du Solide, F-59000 Lille, France.

[5] Department of Chemistry, University of Pennsylvania, Philadelphia, Pennsylvania 19104-6323, United States

Section 1 The Crystal and Magnetic Structure of the $CeMnAsO_{1-x}F_x$ phases

Supplementary Table 1. Refined cell parameters, agreement factors, and atomic parameters obtained for $CeMnAsO_{1-x}F_x$ from Rietveld fits to the *P*4/*nmm* structural model from D2B neutron powder diffraction data at 300 K (origin choice no. 2). Ce and As are located at the 2c site (¼, ¼, *z*), Mn at 2b (¾, ¼, ½), and O/F at 2a (¼, ¾, 0). All samples were phase-pure with the exception of $CeMnAsO_{0.925}F_{0.075}$, which contained a small amount of CeOF (~1.5% by volume).

Atom		$CeMnAsO_{1-x}F_x$			
	x	0.000	0.035	0.050	0.075
Ce	z	0.1319(2)	0.1320(2)	0.1326(3)	0.1328(3)
	U_{iso} (Å^2)	0.0043(6)	0.0048(6)	0.0038(9)	0.0043(7)
Mn	U_{iso} (Å^2)	0.0050(6)	0.0070(5)	0.0064(8)	0.0064(6)
	μ_B(z)	2.59(3)	2.49(3)	2.52(3)	2.46(3)
As	z	0.6713(2)	0.6713(2)	0.6716(3)	0.6714(2)
	U_{iso} (Å^2)	0.0067(5)	0.0065(4)	0.0062(7)	0.0069(5)
O/F	U_{iso} (Å^2)	0.0051(5)	0.0052(4)	0.0048(7)	0.0051(5)
	a (Å)	4.09196(1)	4.09068(4)	4.09012(6)	4.09111(5)
	c (Å)	8.9717(1)	8.9674(1)	8.9644(2)	8.9679(2)
	χ^2	1.22	1.70	1.10	1.32
	R_P (%)	3.24	3.39	3.24	3.60
	R_{WP} (%)	4.09	4.44	4.09	4.67
	V (Å^3)	150.223(2)	150.057(5)	149.966(8)	150.097(7)

Supplementary Table 2. Selected bond lengths and angles for $CeMnAsO_{1\text{-}x}F_x$ obtained from Rietveld fits to the *P*4/*nmm* structural model from D2B neutron diffraction data at 290 K.

x	**0**	**0.035**	**0.05**	**0.075**
**Bond length (Å)**				
Ce–O/F	2.3636(10)	2.3633(9)	2.3653(14)	2.3670(12)
Mn–As	2.5589(10)	2.5577(9)	2.5590(14)	2.5588(12)
Ce–As	3.3896(14)	3.3879(13)	3.3833(20)	3.3840(17)
Mn–Mn	2.89345(1)	2.89255(3)	2.89215(4)	2.89285(4)
**Bond angles (°)**				
α_1 Ce–O/F–Ce	119.91(8)	119.87(7)	119.68(12)	119.58(10)
α_2 Ce–O/F–Ce	104.52(4)	104.54(3)	104.62(5)	104.67(4)
α_1 As–Mn–As	111.14(3)	111.13(3)	111.18(4)	111.16(4)
α_2 As–Mn–As	106.17(6)	106.20(6)	106.10(9)	106.15(7)

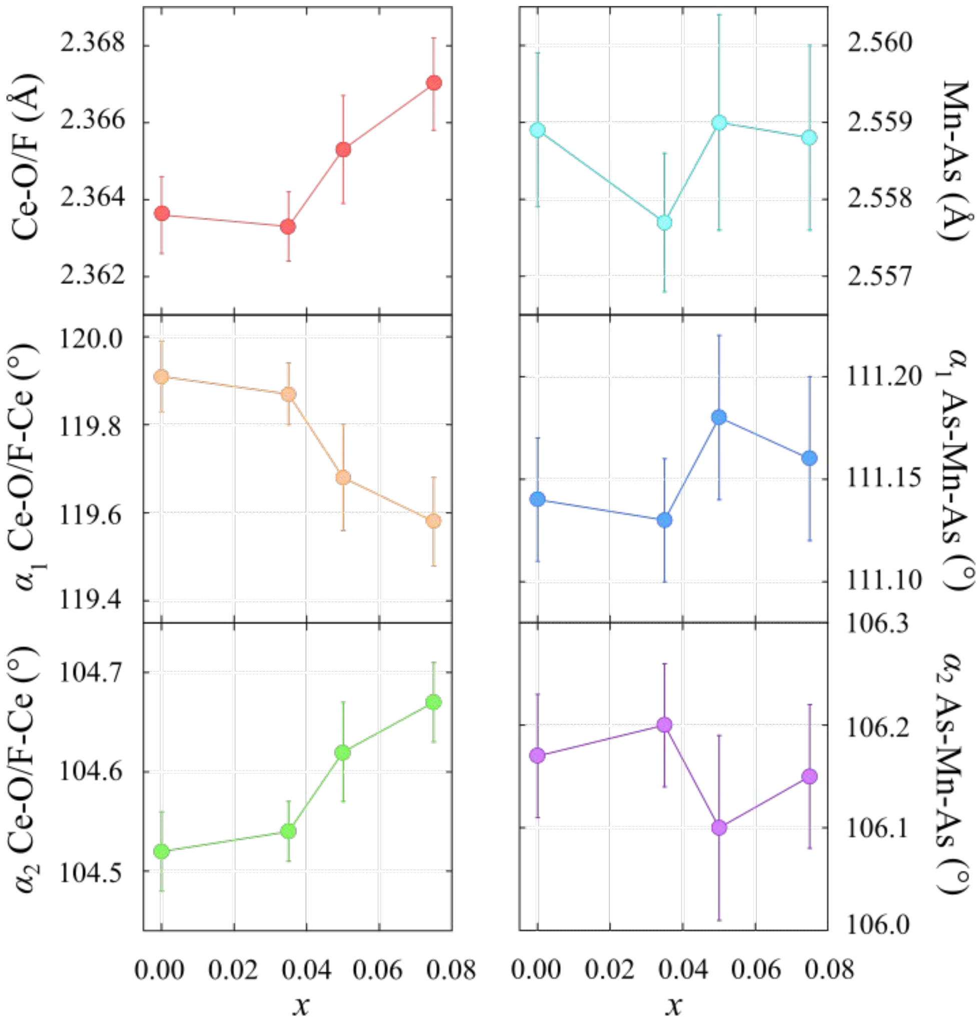

Supplementary Figure 1. Structural trends observed in the Ce(O/F) and MnAs tetrahedral layers across the $CeMnAsO_{1-x}F_x$ series at 290 K. Bond angles are labelled according to Figure 1b.

At ~ 10 K, the Ce-As distance reduces from 3.3779(13) Å for x = 0 to 3.3753(15) Å for x = 0.075 (Supplementary Table 4). The MnAs layers do not display any clear trends upon varying x.

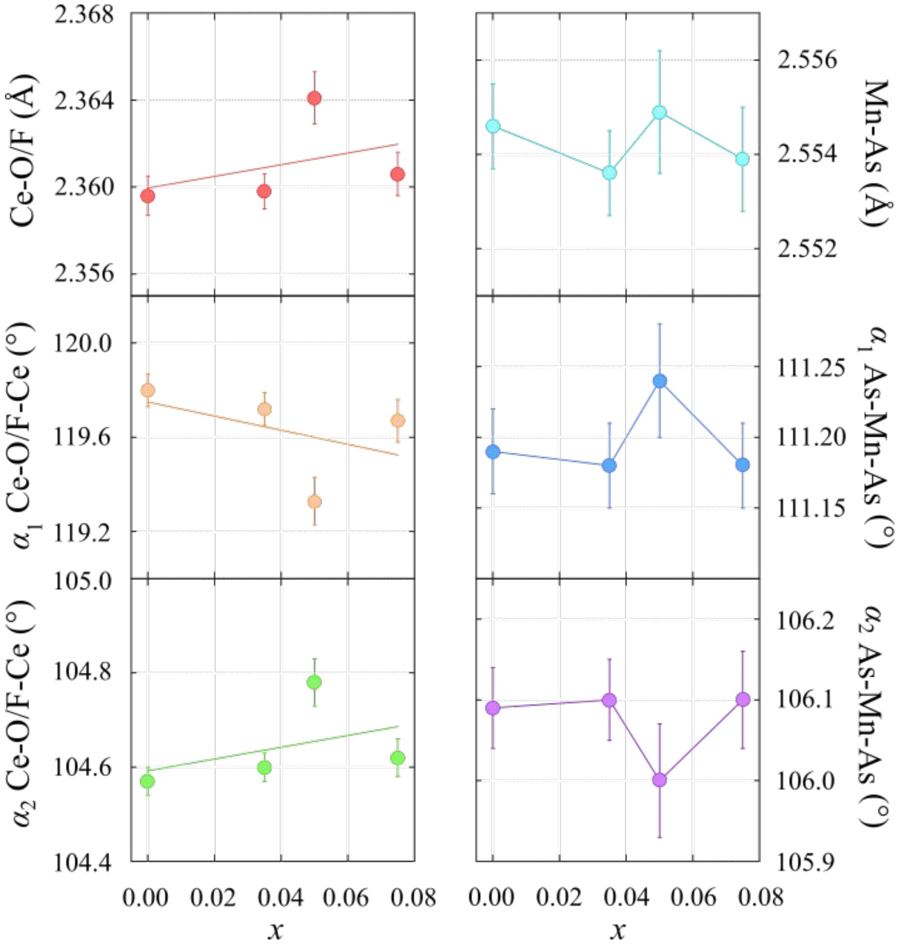

Supplementary Figure 2. Structural trends observed in the Ce(O/F) and MnAs tetrahedral layers across the $CeMnAsO_{1-x}F_x$ series at temperatures < 10 K. Bond angles are labelled according to Figure 3b.

Supplementary Table 3. Refined cell parameters, agreement factors, and atomic parameters obtained for $CeMnAsO_{1-x}F_x$ from Rietveld fits to the *P*4/*nmm* structural model from D2B neutron powder diffraction data at T < 10 K (origin choice no. 2). Ce and As are located at the 2c site (¼, ¼, *z*), Mn at 2b (¾, ¼, ½), and O/F at 2a (¼, ¾, 0).

**Atom**		**$CeMnAsO_{1-x}F_x$**			
	x	**0.000**	**0.035**	**0.050**	**0.075**
**Ce**	*z*	0.1323(2)	0.1325(2)	0.1336(3)	0.1326(2)
	U_{iso} (Å^2)	0.0006(6)	0.0028(5)	0.0011(8)	0.0008(7)
	moment	1.16(4)	1.06(3)	0.97(5)	0.99(5)
**Mn**	U_{iso} (Å^2)	0.0011(5)	0.0020(4)	0.0007(7)	0.0012(6)
	moment	3.65(3)	3.65(3)	3.66(4)	3.64(4)
**As**	*z*	0.6717(2)	0.6717(2)	0.6720(2)	0.6717(2)
	U_{iso} (Å^2)	0.0011(4)	0.0014(4)	0.0006(6)	0.0008(5)
**O/F**	U_{iso} (Å^2)	0.0021(4)	0.0021(4)	0.0019(6)	0.0019(5)
	a (Å)	4.08280(5)	4.08154(4)	4.08079(5)	4.08190(5)
	c (Å)	8.9459(1)	8.9418(1)	8.9378(2)	8.9420(2)
	χ^2 (%)	1.33	1.84	1.16	1.38
	R_P (%)	3.33	3.60	3.34	3.76
	R_{WP} (%)	4.34	4.70	4.28	4.91
	V (Å^3)	149.122(5)	148.961(5)	148.840(7)	148.932(7)

Supplementary Table 4. Selected bond lengths and angles for $CeMnAsO_{1-x}F_x$ obtained from Rietveld fits to the *P*4/*nmm* structural model from D2B neutron diffraction data at low temperature (1.5 – 10 K).

x	0.000	0.035	0.050	0.075
**Bond Length (Å)**				
Ce-O/F	2.3596(9)	2.3598(8)	2.3641(12)	2.3606(10)
Mn-As	2.5546(9)	2.5536(9)	2.5549(13)	2.5539(11)
Ce-As	3.3779(13)	3.3758(12)	3.3681(17)	3.3753(15)
Mn-Mn	2.88697(3)	2.88608(3)	2.88555(4)	2.88634(3)
**Bond Angles (°)**				
α_1 Ce-O/F-Ce	119.80(7)	119.72(7)	119.33(10)	119.67(9)
α_2 Ce-O/F-Ce	104.57(3)	104.60(3)	104.78(5)	104.62(4)
α_1 As-Mn-As	111.19(3)	111.18(3)	111.24(4)	111.18(3)
α_2 As-Mn-As	106.09(5)	106.10(5)	106.00(7)	106.10(6)

Section 2 Magnetic Properties and Thermal Expansion

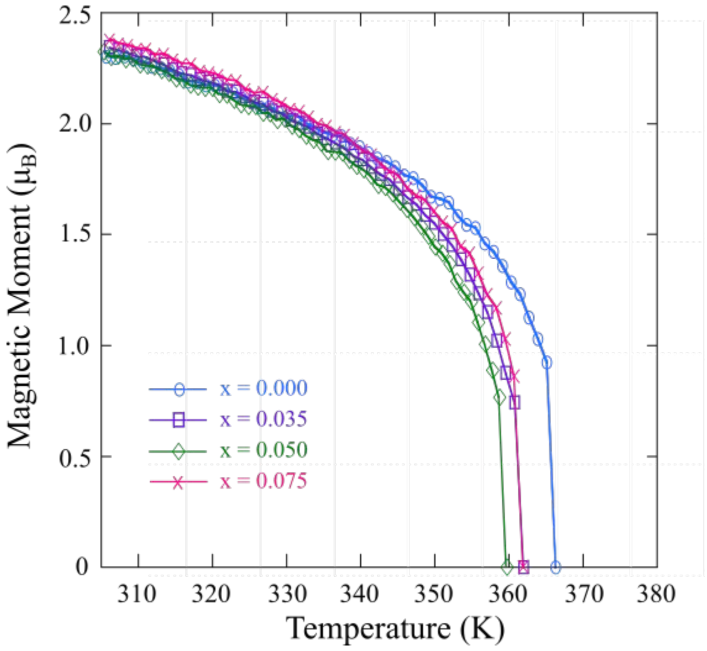

Supplementary Figure 3. Temperature variation of the refined Mn^{2+} magnetic moments for the $CeMnAsO_{1-x}F_x$ series.

In CeMnAsO, the Mn^{2+} moments align antiferromagnetically below 367 K [19]. Below 34 K (T_{SR}) the Ce spins align antiferromagnetically with spins aligned parallel to the basal plane.

This results in a spin reorientation of the Mn^{2+} moments from aligning parallel to *c* to orienting parallel to the basal plane. Variable temperature neutron diffraction patterns of $CeMnAsO_{1-x}F_x$, recorded on the high intensity D20 diffractometer show that there are no significant changes in the magnetic structure at T_{II} so that magnetic ordering is also not responsible for the electronic transition at T_{II}. Only a small reduction in the Mn magnetic transition temperature, T_{Mn}, is observed from 367 K to 359 K upon F^- doping (Supplementary Figure 3).

Thermal Expansion

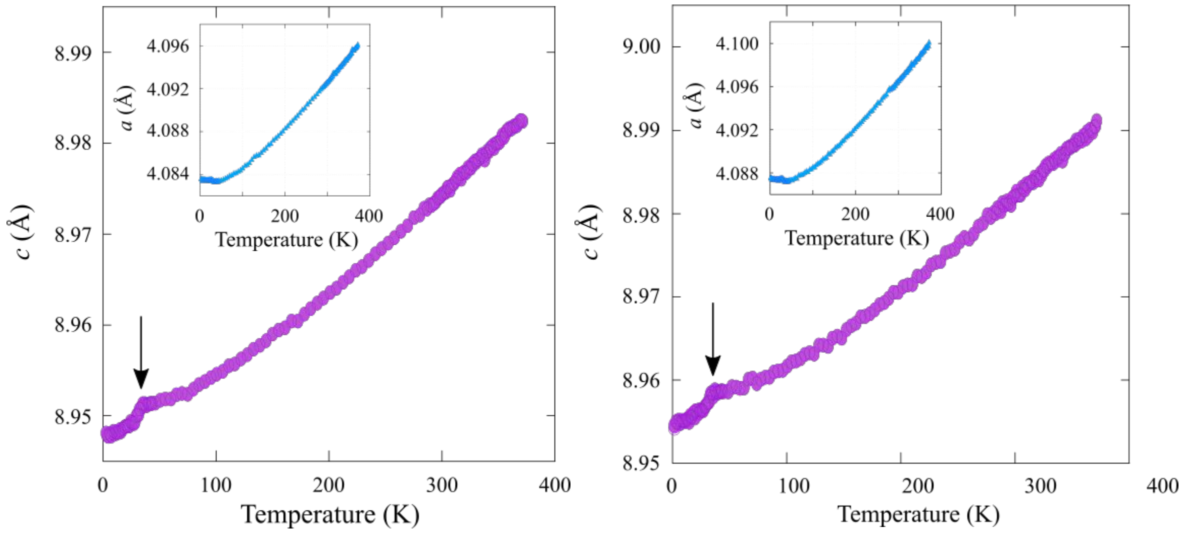

Supplementary Figure 4. Variable temperature plot of the *c* lattice parameter determined from Rietveld fits to the *P*4/*nmm* structural model from D20 neutron diffraction data for $CeMnAsO_{0.965}F_{0.035}$ (left) and $CeMnAsO_{0.925}F_{0.075}$ (right). The variation of the *a* lattice parameter is shown in the inset. The arrow highlights the magnetostriction observed at T_{SR}.

Data were collected on the high intensity D20 diffractometer at the ILL between 1.5 K and 380 K for x = 0.035 and 0.075 and between 300 – 380 K for x = 0.00 and 0.05 due to time constraints. The linear coefficients of thermal expansion were calculated by fitting the temperature dependence of the lattice parameters to a third order polynomial, separately from 50 K - 300 K and 10 K - 30 K (above and below T_{SR}). The coefficients of thermal expansion all begin to plateau above 200 K, consistent with the Debye temperature of 230(10) K. Above T_{SR}, the elastic properties remain anisotropic, such that there is larger thermal expansion along *c*. The anisotropy ratio above T_{SR}, $\alpha(c)/\alpha(a)$, = 1.08(3) 1.11(2), 1.18(3), 1.20(3) and 1.25(5) for x = 0.00, 0.035, 0.05, 0.075 and $Ce_{0.96}MnAsO_{0.95}F_{0.05}$ respectively.

The thermal anisotropy ratio is shown to increase with both x in $CeMnAsO_{1-x}F_x$ and y in $Ce_yMnAsO_{0.95}F_{0.05}$. We hypothesise that this is a result of enhanced interlayer electronic coupling as the Ce-As the inter-layer distance reduces. In further corroboration, changes to the single ion anisotropy of Mn can be ruled out, as the amount of localised charge on the Mn (as determined by the low temperature ordered moment from the neutron diffraction refinements) is, within error, unchanged by F^- content [1]. Changes to the single ion anisotropy of Ce can also be ruled out. A combination of density functional theory (DFT) calculations and neutron diffraction results on the $CeMnAsO_{1-x}F_x$ series have previously shown that upon doping by substitution of F^- for O^{2-}, the Ce 4f band is destabilised and Ce^{3+} is partially oxidised to isotropic Ce^{4+} [1] which should hence *decrease* the anisotropy.

Section 3 Heat Capacity Modelling

To further investigate the interlayer coupling in $CeMnAsO_{1-x}F_x$, heat capacity measurements were recorded for $CeMnAsO_{0.965}F_{0.035}$ and $Ce_{0.96}MnAsO_{0.95}F_{0.05}$. A low temperature Schottky contribution to the heat capacity is observed below T_{SR} (Supplementary Figure 5). This has previously been reported for CeMnAsO [2], where the heat capacity data was fit well with a Schottky term consistent with a Ce^{3+} concentration of 0.965 and a splitting energy of 41 K. We modelled the heat capacity data for $CeMnAsO_{0.965}F_{0.035}$ and $Ce_{0.96}MnAsO_{0.95}F_{0.05}$ with a Schottky term with a Ce^{3+} concentration of 0.84(4) and 0.76(4) per formula unit respectively, consistent with the partial oxidation of Ce^{3+} to Ce^{4+} as observed from the magnetic neutron refinements [1].

The heat capacities of $CeMnAsO_{0.965}F_{0.035}$ and $Ce_{0.96}MnAsO_{0.95}F_{0.05}$ were modelled by a linear combination of Einstein and Debye terms, with Schottky contributions used to describe the low temperature magnetic heat capacity. Thus the overall model used was:

$$C(T) = a_1 C_{D1}(T) + a_2 C_{D2}(T) + bC_E(T) + C_{Schottky1}(T) + C_{Schottky2}(T)$$

$C_D(T)$ represents a Debye function, and $C_E(T)$ represents an Einstein function, where the constraints were used that $a_1+a_2+b=1$ to make sure the total phonon heat capacity saturated at 3R.

The Debye function used for Debye contribution *n* was:

$$C_{Dn}(T) = 9Nk_B\left(\frac{T}{\theta_{Dn}}\right)^3 \int_0^{\theta_{Dn}/T} \frac{x^4 e^x}{(e^x-1)^2} dx$$

Where N is the number density of atoms, k_B is the Boltzmann constant, and θ_{Dn} is the Debye temperature for contribution n.

The Einstein function used for that contribution was:

$$C_E(T) = 3Nk_B(\frac{\theta_E}{T})^2 \frac{e^{\frac{\theta_E}{T}}}{(e^{\frac{\theta_E}{T}} - 1)^2}$$

Where θ_E is the Einstein temperature of the localised oscillator.

The Schottky function that was used for those contributions (one for the splitting of the Ce doublet and one for remnant localised spins at very low temperatures) was

$$C_{Schottky}(T) = Nk_B(\frac{\Delta}{T})^2 \frac{e^{\frac{\Delta}{T}}}{(e^{\frac{\Delta}{T}} + 1)^2}$$

Where $\Delta = E_S/k_B$ and E_S is the energy difference between the two levels, and N is the number density of the atom or defect that generates those energy levels.

$CeMnAsO_{0.965}F_{0.035}$

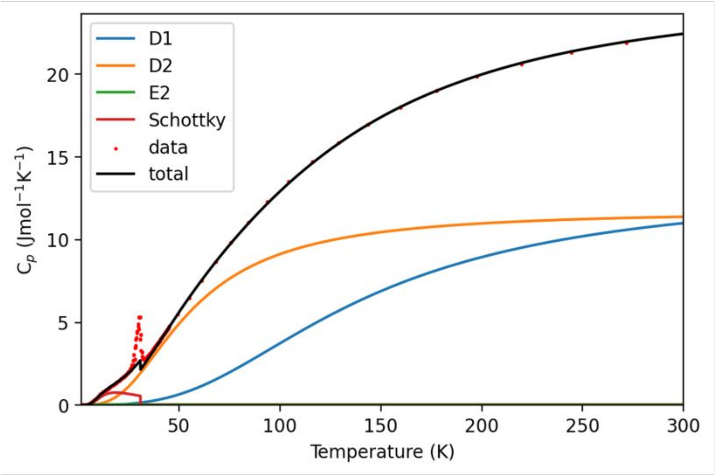

$Ce_{0.96}MnAsO_{0.95}F_{0.05}$

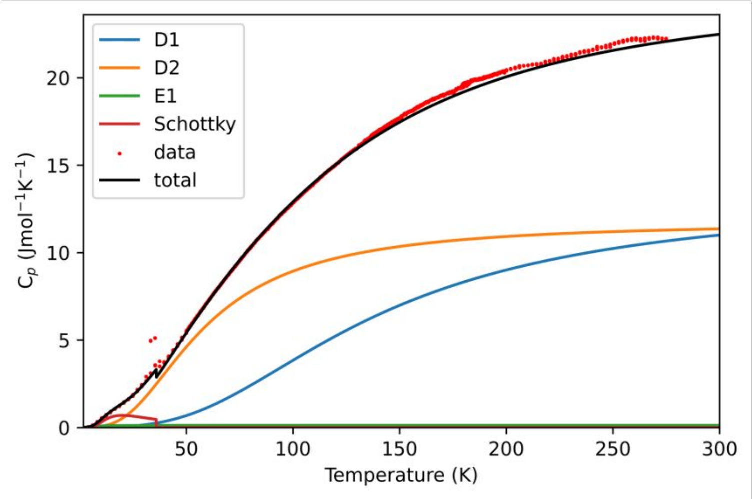

Supplementary Figure 5. Heat capacity (C_P) versus temperature for $CeMnAsO_{0.965}F_{0.035}$ and $Ce_{0.96}MnAsO_{0.95}F_{0.05}$ showing the total model as well as the two Debye contributions (D1 and D2) along with the Schottky contribution. The Einstein and low temperature Schottky contributions matter most at very low temperatures.

To separate the phonon contribution from the Schottky contribution to the heat capacity, we modelled the experimental heat capacity with a linear combination of phonon and Schottky contributions. This fit is shown in Supplementary Figure 6. The phonon contribution to the heat capacity for $CeMnAsO_{0.965}F_{0.035}$ is described well by a linear combination of two Debye terms,

with T_{D1} = 580(10) K and T_{D2} = 230(10) K. The average Debye temperature, including the respective weighting of these terms, is 414(14) K, which is associated with an average Debye velocity of 3.7(2) x 10^3 m/s. This velocity is within error of the average speed of sound calculated for LaFeAsO [3, 4]. The heat capacity could not be satisfactorily fit with only one Debye term – the use of two Debye terms is often necessary for complex systems with large mass contrast, due to the splitting of the phonon density of states into two distinct regions[5]. A comparison of the parameters used to model the heat capacities are show in Supplementary Table 5 below. All uncertainties were estimated by the amount the parameter needed to change to produce a qualitatively worse fit, and are likely conservative over-estimates.

Supplementary Table 5 Fitting parameters from the variable temperature heat capacity data.

Material	T_{D1} (K)	T_{D2} (K)	T_E (K)	Schottky 1	Schottky 2
$CeMnAsO_{0.965}F_{0.035}$	580(10)	230(10)	20(1)	E_S/k_B = 45(1) K<br>N = 0.21(1)	E_S/k_B = 6(1) K<br>N = 0.00005(1)
$Ce_{0.96}MnAsO_{0.95}F_{0.05}$	570(1)	240(10)	20(1)	E_S/k_B = 48(2) K<br>N = 0.19(1)	E_S/k_B = 6(1) K<br>N = 0.00006(1)

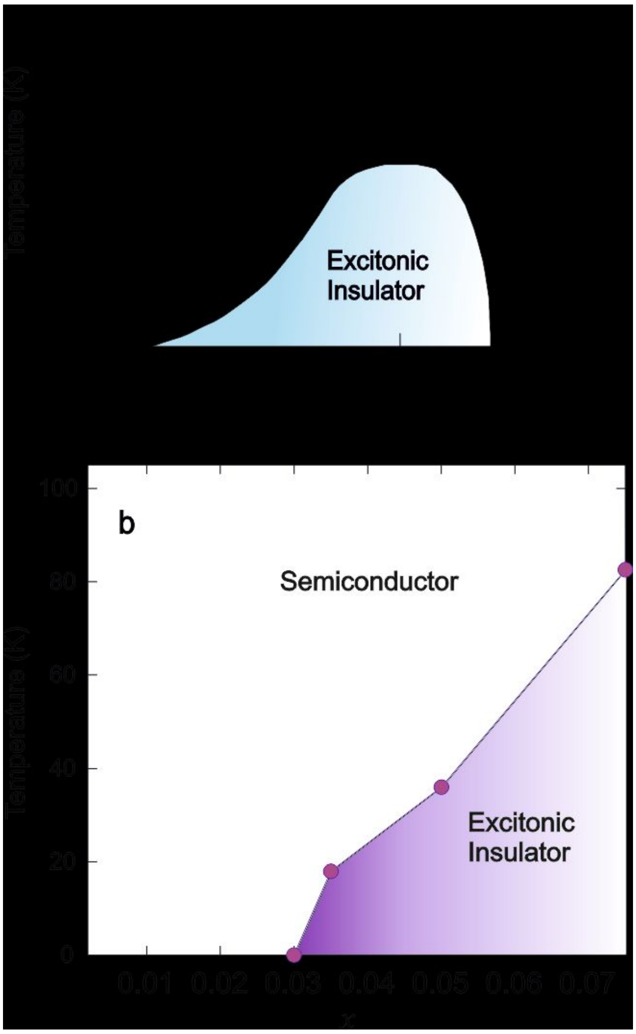

Supplementary Figure 6. Comparison of the phase diagram of $CeMnAsO_{1\text{-}x}F_x$ with that of an excitonic insulator. (a) Electronic phase diagram of an excitonic insulator as a function of band gap, E_g, assuming a constant exciton binding energy. (b) The electronic phase diagram of $CeMnAsO_{1\text{-}x}F_x$ which exhibits a semiconductor-insulator phase transition with a clear dependence on the doping value, x.

References

1. Wildman, E. J. et. al. Observation of an Exotic Insulator to Insulator Transition upon Electron Doping the Mott Insulator CeMnAsO. *Nature Commun.* **14**, 7037 (2023).

2. Stokes, H. T., Hatch, D. M. & Campbell, B. J. ISODISTORT, ISOTROPY Software Suite, iso.byu.edu.

3. Campbell, B. J., Stokes, H. T., Tanner, D. E. & Hatch, D. M. "ISODISPLACE: An Internet Tool for Exploring Structural Distortions." J. Appl. Cryst. **39**, 607-614 (2006).
